# Supplementary material for: Risk factors analysis for neglected human rickettsioses in rural communities in Nan province, Thailand: A community-based observational study along a landscape gradient
Source: PLoS Negl Trop Dis. 2022 Mar 23;16(3):e0010256. doi: 10.1371/journal.pntd.0010256 (PMC8979453; doi:10.1371/journal.pntd.0010256)
Supplement: S1 Table — (DOCX) [file pntd.0010256.s001.docx]

**S1 Table.** Demographic data of each village in Saen Thong Sub-district, Tha Wang Pha District, Nan Province, Thailand.

| **Parameter** | **Total** | **Village No.1**  **Na Noon** | **Village No.2**  **Na Sai** | **Village No.3**  **Pho** | **Village No.8**  **Hae** | **Village No.4**  **Huak** | **Village No.5**  **Nam Krai** | **Village No.6**  **Huay Muang** | **Village No.7**  **Santisuk** |
| --- | --- | --- | --- | --- | --- | --- | --- | --- | --- |
| **Number of households*** | 1,083 | 247 | 120 | 109 | 119 | 210 | 86 | 128 | 64 |
| **Number of tested house (%)** | 524 (48.3%) | 104 (42.1%) | 74 (61.6%) | 60 (55.0%) | 46 (38.6%) | 93 (44.3%) | 43 (50.0%) | 75 (58.6%) | 29 (45.3%) |
| **Total population*** | 4,145 | 807 | 446 | 385 | 415 | 890 | 418 | 510 | 274 |
| **Landscape type** |  | Lowland urbanized  area | Lowland urbanized  area | Lowland urbanized  area | Lowland urbanized  area | Upland forested area | Upland forested area | Upland forested  area | Upland forested  area |
| **Gender (%)*** |  |  |  |  |  |  |  |  |  |
| Number of males | 2,090 (50.4%) | 378 (46.8%) | 234 (52.5%) | 195 (50.6%) | 225 (54.2%) | 465 (52.2%) | 213 (50.9%) | 258 (50.6%) | 122 (44.5%) |
| Number of females | 2,055 (49.6%) | 429 (53.2%) | 212 (47.5%) | 190 (49.4%) | 190 (45.8%) | 425 (47.8%) | 205 (49.1%) | 252 (49.4%) | 152 (55.5%) |
| **Age group (%)*** |  |  |  |  |  |  |  |  |  |
| Number of children (0-17) | 631 (15.2%) | 101 (12.5%) | 62 (13.9%) | 47 (12.2%) | 57 (13.7%) | 151 (16.9%) | 79 (18.9%) | 91 (17.8%) | 43 (15.7%) |
| Number of young adults  (18-35) | 958 (23.1%) | 160 (19.8%) | 114 (25.6%) | 86 (22.4%) | 97 (23.4%) | 204 (23.0%) | 103 (24.6%) | 128 (25.1%) | 66 (24.1%) |
| Number of adult (36-60) | 1,706 (41.2%) | 300 (37.3%) | 183 (41.0%) | 172 (44.6%) | 166 (40.0%) | 375 (42.1%) | 171 (40.9%) | 220 (43.1%) | 119 (43.4%) |
| Number of elderly (>60) | 850 (20.5%) | 246 (30.4%) | 87 (19.5%) | 80 (20.8%) | 95 (22.9%) | 160 (18.0%) | 65 (15.6%) | 71 (14.0%) | 46 (16.8%) |
| **Career (%)*** |  |  |  |  |  |  |  |  |  |
| Farmer | 1,897 (45.7%) | 347 (42.9%) | 164 (36.7%) | 191 (49.6%) | 171 (41.2%) | 141 (15.9%) | 309 (73.9%) | 376 (73.7%) | 198 (72.3%) |
| Non-farmer | 1,698 (41.0%) | 460 (57.1%) | 282 (63.3%) | 194 (50.4%) | 244 (58.8%) | 199 (22.3%) | 109 (26.1%) | 134 (26.3%) | 76 (27.7%) |
| No information | 550 (13.3%) | - | - | - | - | 550 (61.8%) | - | - | - |
| **Number of participants tested for rickettsiosis exposure (% participation)** | 824  (19.8%) | 161  (19.9%) | 113  (25.3%) | 98  (25.5%) | 71  (17.1%) | 141  (15.8%) | 64  (15.3%) | 122  (23.9%) | 54  (19.7%) |

* Registered in the database of the local Health Promoting Hospital
