# Supplementary figures and images for: Risk factors analysis for neglected human rickettsioses in rural communities in Nan province, Thailand: A community-based observational study along a landscape gradient
Source: PLoS Negl Trop Dis. 2022 Mar 23;16(3):e0010256. doi: 10.1371/journal.pntd.0010256 (PMC8979453; doi:10.1371/journal.pntd.0010256)

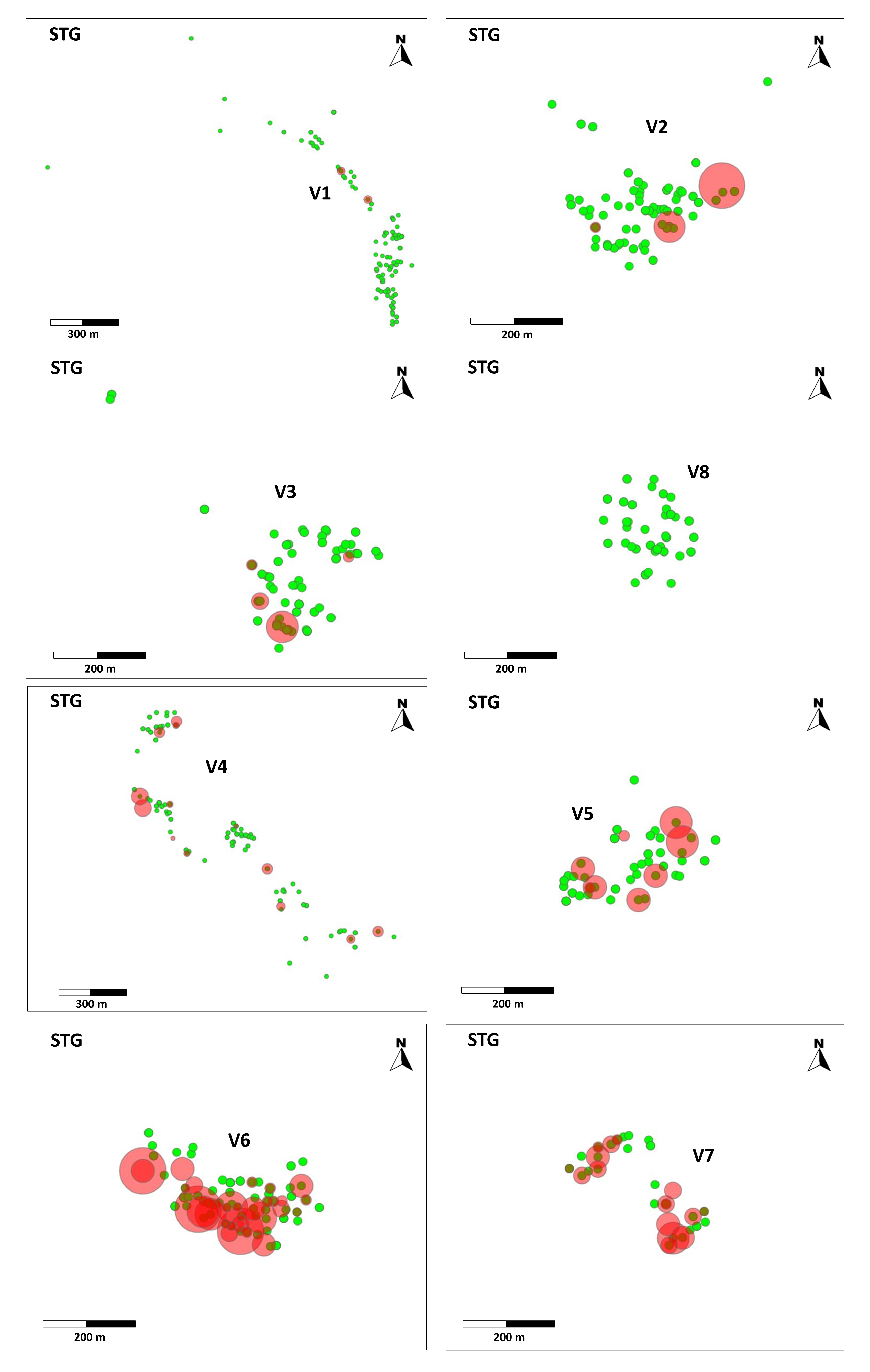

Supplement: S1 Fig — Green and red circles indicate sero-negativity and sero-positivity of STG, respectively. Size of the red circles indicate level of STG positivity (IFA titers). (TIF) [file pntd.0010256.s002.TIF]

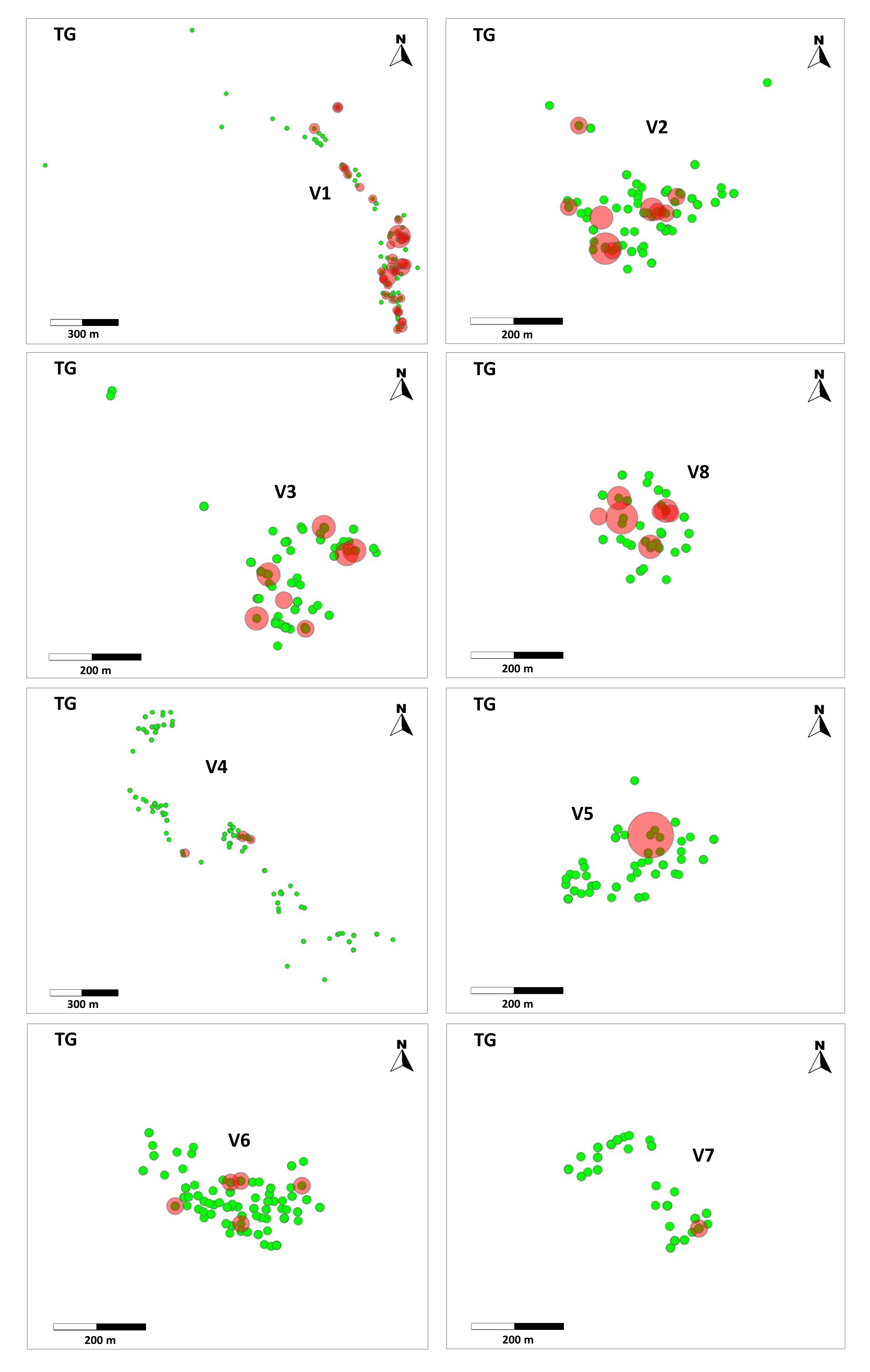

Supplement: S2 Fig — Green and red circles indicate sero-negativity and sero-positivity of TG, respectively. Size of the red circles indicate level of TG positivity (IFA titers). (TIF) [file pntd.0010256.s003.TIF]

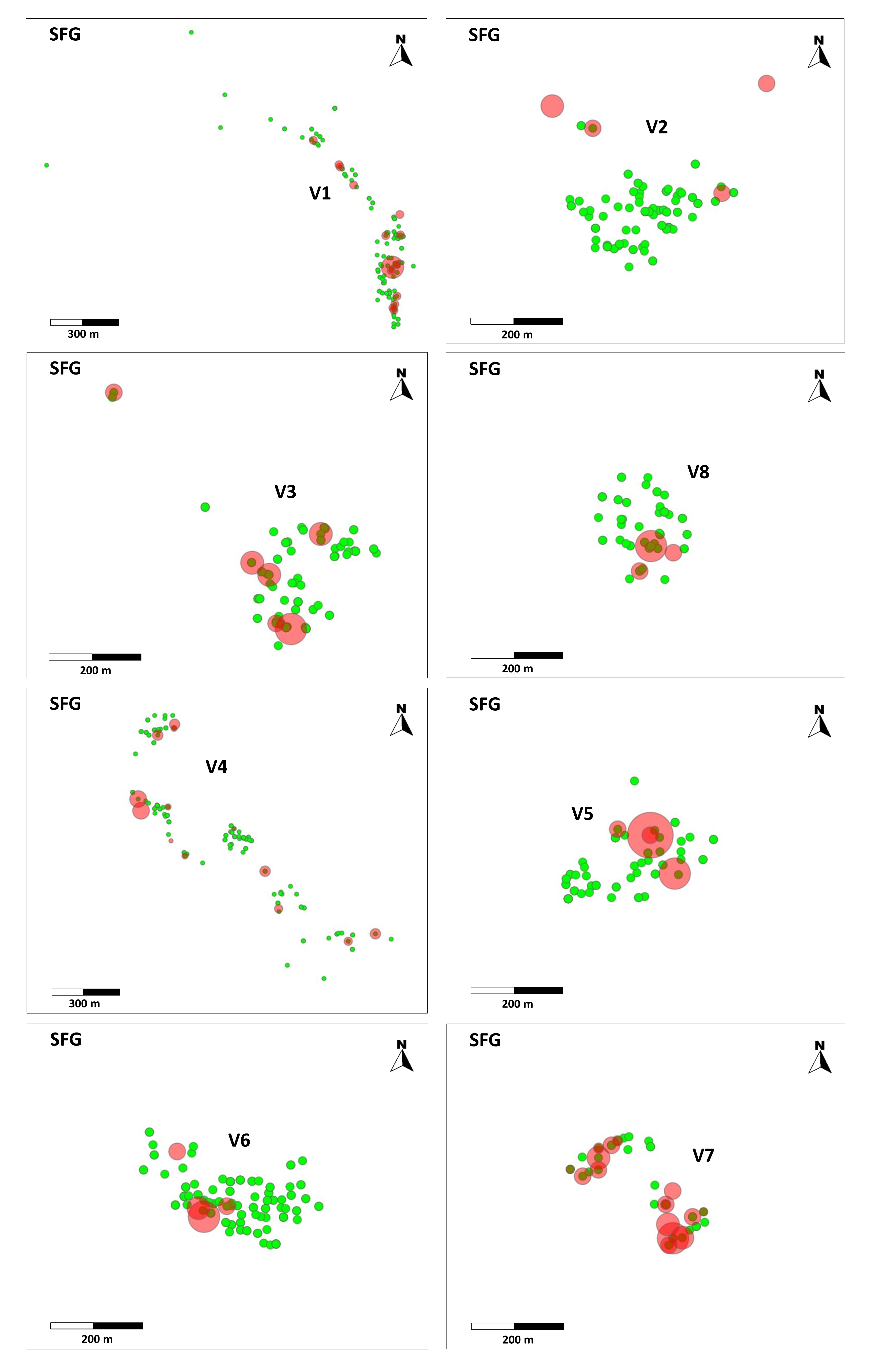

Supplement: S3 Fig — Green and red circles indicate sero-negativity and sero-positivity of SFG, respectively. Size of the red circles indicate level of SFG positivity (IFA titers). (TIF) [file pntd.0010256.s004.TIF]
