# Supplementary material for: Risk factors analysis for neglected human rickettsioses in rural communities in Nan province, Thailand: A community-based observational study along a landscape gradient
Source: PLoS Negl Trop Dis. 2022 Mar 23;16(3):e0010256. doi: 10.1371/journal.pntd.0010256 (PMC8979453; doi:10.1371/journal.pntd.0010256)
Supplement: Attachment — Submitted filename: Responses to reviewers_MS_Chaisiri et al 2021_final.docx [file pntd.0010256.s005.docx]

**Author responses to the editor/reviewers comments**

*We are appreciated by your kindness allowing us a great opportunity to revise the manuscript for consideration to publish our work in the PLOS NTDs. We would like to gratefully thank also to the constructive comments and suggestions from you. Here, we carefully read through all comments, and do manuscript revision to our best of knowledge. The revisions in the text regarding to reviewers comments are highlighted in red. Also, please consider our point-to-point responses to the associate editor’s and the reviewers’ comments as the details below.*

**Reviewer's Responses to Questions**
Key Review Criteria Required for Acceptance?
As you describe the new analyses required for acceptance, please consider the following

**Methods**
-Are the objectives of the study clearly articulated with a clear testable hypothesis stated?
-Is the study design appropriate to address the stated objectives?
-Is the population clearly described and appropriate for the hypothesis being tested?
-Is the sample size sufficient to ensure adequate power to address the hypothesis being tested?
-Were correct statistical analysis used to support conclusions?
-Are there concerns about ethical or regulatory requirements being met?

**Reviewer #1:** Methods appear to be fine. I do not have the statistical expertise to critique the methods used, but they seem to be ok at a superficial level.

***Response to the reviewer:*** Thank you very much for your kind and a positive comment.

Reviewer #2: Methodology is well described and objectives are clear. Statistical analysis is correct. No ethical issues

***Response to the reviewer:*** Thank you very much for your kind and a positive comment.

Reviewer #3: Materials and Methods; Pg 7; Line 21 (Blood sample collection)
Please specify the details of the tubes used to collect serum or give the relevant reference.
You could change it like this if needed: Blood samples were collected and processed for rickettsial serology as described previously/elsewhere

***Response to the reviewer:*** Thank you very much for your kind suggestion. We add more details on the procedures and equipment used to collect blood and serum samples. Please consider the addition in page 7 (line 20-22).

--------------------
**Results**
-Does the analysis presented match the analysis plan?
-Are the results clearly and completely presented?
-Are the figures (Tables, Images) of sufficient quality for clarity?

**Reviewer #1:** Results are valid

***Response to the reviewer:*** Thank you very much for your kind and a positive comment.

**Reviewer #2:** The analysis planned and presented matched each other. Results clearly and comprehensively presented. Quality of the images and tables is as per the standard requirements.

***Response to the reviewer:*** Thank you very much for your kind and a positive comment.

**Reviewer #3:** Pg 10; lines 4 & 6, you have mentioned age and occupation could not be assigned in 1.8% and 5.1% respectively. Kindly give a reason/reasons for this. If data was not collected/ or was unclear, you can omit this statement

***Response to the reviewer:*** These individual data were accidentally missing during the interviewing step. So, we add this statement to clarify in the text, please consider at page 10 (line 1-4).

Pg 10; Line 7-11; Regarding individual interviewing ------ keeping these animals at home, respectively. Sentence is not clear (complicated) please re-write in simple words, so that it is easy to understand.

***Response to the reviewer:*** We have revised the sentences, please consider the change at page 10 (line 5-9).

Pg 10 Line14-15: Simplify this sentence

***Response to the reviewer:*** We have revised the sentences, please consider the change at page 10 (line 13-17).

--------------------

**Conclusions**
-Are the conclusions supported by the data presented?
-Are the limitations of analysis clearly described?
-Do the authors discuss how these data can be helpful to advance our understanding of the topic under study?
-Is public health relevance addressed?

Reviewer #1: Conclusions appear to be valid and based on the data.

***Response to the reviewer:*** Thank you very much for your kind and a positive comment.

Reviewer #2: Conclusions are supported by the data. Authors have explained the usefulness of the study with reference to Public health. Few limitations could have been added like need for exploring the burden of rodent and ecto parasites in the two contrasting settings.

***Response to the reviewer:*** We thank the reviewer for this invaluable suggestion. We agree with this, and we add a statement to suggest investigation of rodent-ectoparasite-rickettsiae for further study. Please consider this addition in page 14-15 (line 23-24 and 1-3).

Reviewer #3: (No Response)

--------------------

Editorial and Data Presentation Modifications?

Use this section for editorial suggestions as well as relatively minor modifications of existing data that would enhance clarity. If the only modifications needed are minor and/or editorial, you may wish to recommend “Minor Revision” or “Accept”.

Reviewer #1: The standard of written English is not up to publication level. Suggest review by a native English speaker.

***Response to the reviewer:*** The manuscript has been thoroughly revised in its English writing by a native English speaker.

Introduction, line 14. The mites that appear to transmit Orientia sp in Chile are not of the Trombiculidae family.

***Response to the reviewer:*** We do not agree with the reviewer’s statement. The most recent publication in Chile reported that *Herpetacarus antarctica* are anthropophilic causing human trombiculiasis and serve as vector for scrub typhus in southern Chile. This was proven as the vector of scrub typhus which does not belong to the genus *Leptotrombidium* (Weitzel T, Silva-de la Fuente MC, Martínez-Valdebenito C, Stekolnikov AA, Pérez C, Pérez R, Vial C, Abarca K, Acosta-Jamett G (2021). Novel Vector of Scrub Typhus in Sub-Antarctic Chile: Evidence From Human Exposure, Clinical Infectious Diseases, ciab748, <https://doi.org/10.1093/cid/ciab748>).

In Chiloe island, three chigger genera, i.e., *Herpetacarus* sp., *Quadraseta* sp., and *Paratrombicula* sp. were found infesting on rodents. All of *Orientia*-positive were only found in the Genus *Herpetacarus*, concluding that the genus serves as important vectors and reservoirs of scrub typhus in South America (Acosta-Jamett G, Martı ́nez-Valdebenito C, Beltrami E, Silva-de La Fuente MC, Jiang J, Richards AL, et al. (2020) Identification of trombiculid mites (Acari: Trombiculidae) on rodents from Chiloe ́ Island and molecular evidence of infection with *Orientia* species. PLoS Negl Trop Dis 14(1): e0007619. https://doi.org/10.1371/journal.pntd.0007619).

Certainly, the genus *Herpetacarus* is classified in the Family Trombiculidae. Regarding the reviewer comment’s, we believe that the reviewer probably meant that *Orientia sp* in Chile are transmitted by non-*Leptotrombidium* chiggers.

Material & Methods, line 10/11. What is meant by "no communication problem" ? autism ? poor mobile phone coverage ?

***Response to the reviewer:*** No communicable problem in this situation means to the personal ability to respond the interview and questionnaire session (e.g., dumb, deaf and autism, for instance). We add a statement to emphasize to this point, please consider this addition in page 7 (line 9-11).

I was surprised that only 6 sera (3.1%) showed cross-reactions between TG-SFG in IF serology. In my lab we see a much higher level of cross-reaction.

***Response to the reviewer:*** Thank you to the reviewer for this comment. We are familiar with the concept of TG-SFG cross reaction. However, in our experience, it does not appear to be a significant issue. This may be because of the way we produce our diagnostic reagents and particular *Rickettsia spp.* strains that we use for our antigens. Also, because we screen with ELISA and then titrate positives using IFA, often low-level cross reactions are titrated out following serial dilution of the sample.

p. 12 line 1/2 "(non-exposure to 4.1%)" What does this mean ?

***Response to the reviewer:*** We change from “non-exposure to 4.1%” to “none to 4.1%”.

Table 1. Data is clear but why does village # 8 come after # 3 rather than after # 7 ? It looks untidy.

***Response to the reviewer:*** We categorized these villages regarding their landscape types (No. 1, 2, 3 and 8 are from lowland agriculture landscape; whereas No. 4, 5, 6 and 7 are from upland forested landscape) rather than the order of administrative number of the villages. To make it clearer for readers, Table 1 is modified by adding Lowland agriculture landscape and Upland forested landscape as the subcategory for Village. Please consider this change in Table 1 (page 27).

Reviewer #2: Cut-off titres for ST IgG IFA for epidemiological purposes as opined by few authors may be added.

***Response to the reviewer***: Cut-off titers for ST IgG IFA from other studies have been added in the Results and Discussion, please consider this addition in page 11 (line 2 and line 5).

Reviewer #3: (No Response)

--------------------

**Summary and General Comments**

Use this section to provide overall comments, discuss strengths/weaknesses of the study, novelty, significance, general execution and scholarship. You may also include additional comments for the author, including concerns about dual publication, research ethics, or publication ethics. If requesting major revision, please articulate the new experiments that are needed.

Reviewer #1: An interesting study and well performed with clear data.

***Response to the reviewer:*** Thank you very much for your kind and a positive comment.

Reviewer #2: The study is well planned and meticulously carried out.

***Response to the reviewer:*** Thank you very much for your kind and a positive comment.

Reviewer #3: Authors have conducted a study which expands the body of knowledge regarding the important factors contributing to rickettsial infections in Asia and Thailand in particular. As the methodology is robust, the results and the conclusions derived are reliable. I have a few minor suggestions which are appended as comments. Another suggestion is that the language and grammar though adequate in most places needs more attention
Example: Pg12; Line 24 (going to Pg13; Line 1): Lacking of education is recognized as one of poverty indices.

***Response to the reviewer:*** The manuscript has been thoroughly revised in its English writing by a native English speaker.
