# Supplementary material for: Risk factors analysis for neglected human rickettsioses in rural communities in Nan province, Thailand: A community-based observational study along a landscape gradient
Source: PLoS Negl Trop Dis. 2022 Mar 23;16(3):e0010256. doi: 10.1371/journal.pntd.0010256 (PMC8979453; doi:10.1371/journal.pntd.0010256)
Supplement: Attachment — Submitted filename: Response to the reviewers_resub2.docx [file pntd.0010256.s006.docx]

Kittipong Chaisiri (PhD)

Faculty of Tropical Medicine, Mahidol University,

420/6 Ratchavithi Rd., Ratchathewi,

Bangkok, Thailand 10400

Tel/Fax: +66 (0) 2643 5600

Email address:

[kittipong.cha@mahidol.ac.th](mailto:kittipong.cha@mahidol.ac.th)

[kittipong.chaisiri@gmail.com](mailto:kittipong.chaisiri@gmail.com)

9^th^ February, 2022

**Resubmission of the manuscript number PNTD-D-21-01304R1**

Editor of PLOS Neglected Tropical Diseases,

On behalf of the authors, we would like to thank the editor and the three reviewers for this great opportunity to consider our manuscript to be published in the PLoS NTDs. We've considered the valuable comments from you, particularly the two minor comments from the Reviewer #1. Here, we carefully read through the suggestions, and do manuscript revision to our best of knowledge. The revisions in the text regarding to the reviewer’s comments are highlighted in red with a track change notification. Also, please consider our point-to-point responses to your comments as the details below.

**Reviewer #1:** line 9 of p7 states "age over 9 years" but line 12 states "children 7-9 years old". Which statement is correct? Currently the 2 statements are inconsistent.

**Authors’ response:** Thank you very much for your comment. We have corrected this typo to keep the statements consistency. Please consider the change in page 7, line 12.

**Reviewer #1:** line 22 of p7 states "rpm". This is an inappropriate representation of centrifugal force as it can vary with the diameter of the centrifuge rotor. It should always be expressed as " x g ".

**Authors’ response:** We do agree with the reviewer comment. As we did centrifugation of blood specimens with a 10 cm-radius rotor machine, so the centrifugation force at 2,795 x g is written in the text instead of 5,000 rpm. Please consider the change in page 7 , line 22.

Thank you very much for your time and consideration. Please let us know, if any further information required. We are looking forward to hear your decision.

Yours sincerely,

Kittipong Chaisiri

Co-corresponding author
